# Supplementary material for: The human leukemia virus HTLV-1 alters the structure and transcription of host chromatin in cis
Source: eLife. 2018 Jun 26;7:e36245. doi: 10.7554/eLife.36245 (PMC6019074; doi:10.7554/eLife.36245)
Supplement: Supplementary file 1. — Extended data on clones shown in Table 1. All subjects are HTLV-1 carriers with HAM/TSP, except for HAY who is an asymptomatic carrier of HTLV-1. tax expression of ‘high’ or ‘low’ denotes whether the frequency of plus-strand viral transcripts was higher or lower than the median, respectively. [file elife-36245-supp1.docx]

Supplementary File 1 – Clones and integration sites used in this work

| Subject code | clone | Integration site | | | Tax expression |
| --- | --- | --- | --- | --- | --- |
|  |  | Chromosome | Position | Strand |  |
| TBJ | 3.60 | chr4 | 70567285 | Fwd | High |
|  | 3.83 | chr14 | 46204950 | Fwd | Low |
| TCX | 8.13 | chr16 | 53601059 | Fwd | Low |
|  | 8.8 | chr13 | 76099820 | Fwd | High |
| TCT | 10.1 | chr12 | 42634117 | Fwd | Low |
| TBW | 11.50 | chr19 | 28282587 | Rev | High |
|  | 11.63 | chr19 | 33829548 | Fwd | High |
|  | 11.65 | chr3 | 76494270 | Fwd | Low |
|  | 13.5 | uninfected | | | NA |
| TBX | TBX4B | chr22 | 44323198 | Fwd | High |
| HAY | 6.25 | chr1 | 186478980 | Fwd | Low |
|  | 6.3 | uninfected | | | NA |
