# Supplementary material for: The human leukemia virus HTLV-1 alters the structure and transcription of host chromatin in cis
Source: eLife. 2018 Jun 26;7:e36245. doi: 10.7554/eLife.36245 (PMC6019074; doi:10.7554/eLife.36245)
Supplement: Supplementary file 2. — In the conventional protocol (A), after digesting the crosslinked chromatin with the first restriction enzyme (1 st RE) and ligating the free ends, the DNA was digested with a second restriction enzyme (second RE) followed by religation and inverse PCR to amplify viewpoint (VP)-linked genomic regions. In q4C, we modified the 4C protocol (Krijger and de Laat, 2016) by applying the approach used in our previously described linker-mediated (LM)-PCR protocol (Gillet et al., 2011) for identifying and quantifying proviral integration sites. In q4C, instead of the secondary restriction enzyme, sonication is used to process DNA circles. Linkers with a 6 bp specific tag was added to sonicated DNA. The end of the VP and a fragment of genomic DNA were amplified by LM-PCR. In this example, three ligation events occurred between the VP (red) and a genomic region (green) at the ligation site I and one event at the ligation site II (yellow). Because the DNA shear site is (approximately) random, the amplicon from each cell has a different shear site. The abundance of ligation events at each respective ligation site is quantified by counting the number of different shear sites. [file elife-36245-supp2.pptx]

## Slide 1
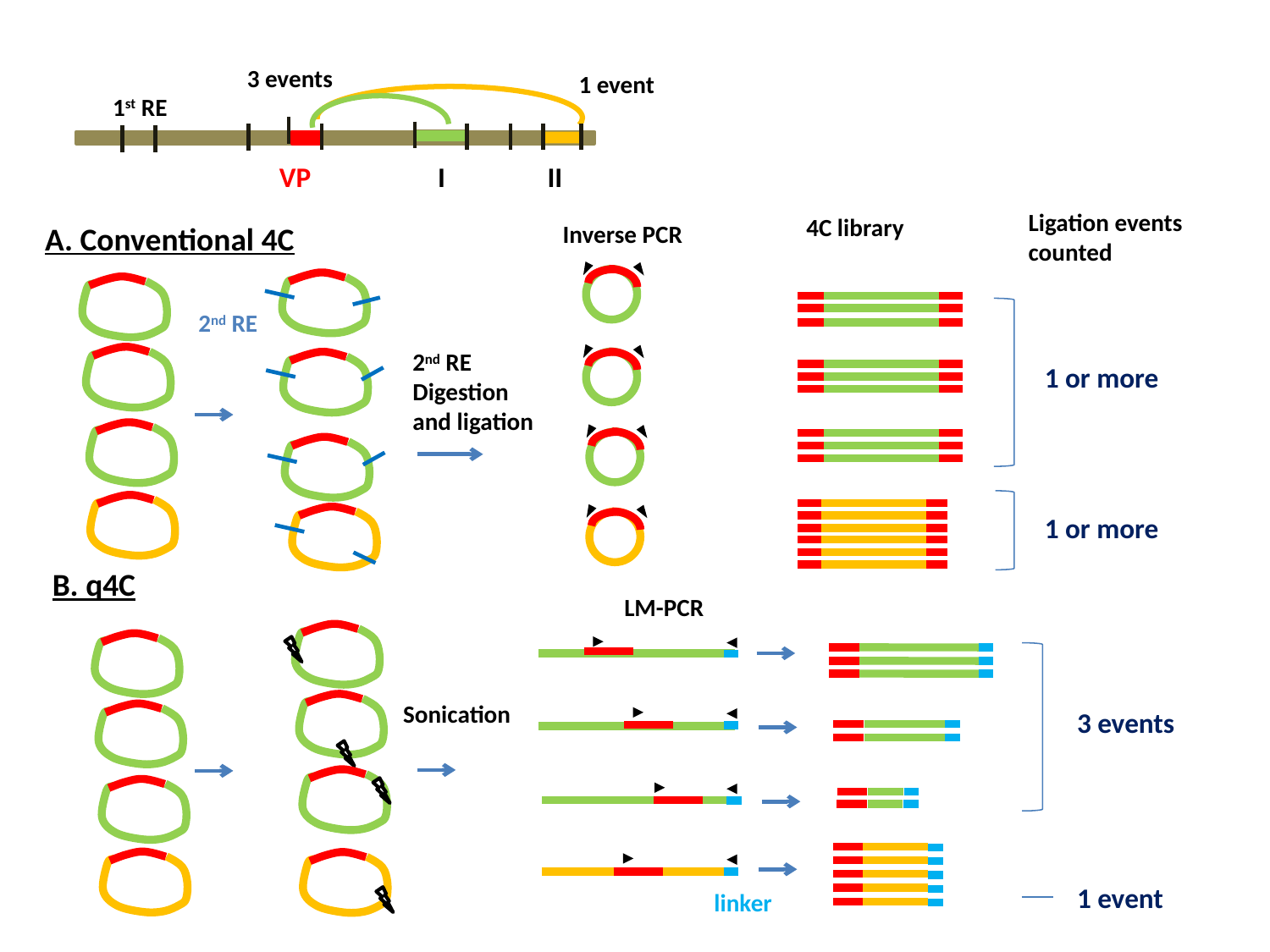

1 event
3 events
1st RE
VP
I
II
Ligation events counted
4C library
Inverse PCR
A. Conventional 4C
2nd RE
2nd RE
Digestion
and ligation
1 or more
1 or more
B. q4C
LM-PCR
Sonication
3 events
1 event
linker
