# Supplementary material for: The human leukemia virus HTLV-1 alters the structure and transcription of host chromatin in cis
Source: eLife. 2018 Jun 26;7:e36245. doi: 10.7554/eLife.36245 (PMC6019074; doi:10.7554/eLife.36245)
Supplement: Supplementary file 3. — Summary of main steps in the analysis steps of q4C data. See Materials and methods for details. [file elife-36245-supp3.pdf]

q4C Data analysis steps

	• Data (Fastq) from sequencing facility: demultiplexed by barcode
Trim	• remove low quality reads and linker sequences
Digest	• digest NlaIII in silico fragments to break composite reads
Align	• align against combined reference (human + viral)
Extraction	• identify and quantify ligation sites
Filter	• remove barcode errors, decrease noise by selecting convergent reads
Peak calling	• identify reproducible contacts.
